# Remarks on reporting and recording consistent with the ICRU Reference Dose

**DOI:** 10.1186/1748-717X-4-44

**Published:** 2009-10-14

**Authors:** Klaus Bratengeier, Markus Oechsner, Mark Gainey, Michael Flentje

**Affiliations:** 1 Klinik und Poliklinik für Strahlentherapie, University of Würzburg Josef-Schneider-Str. 11, 97080 Würzburg, Germany

## Abstract

**Background:**

ICRU 50/62 provides a framework to facilitate the reporting of external beam radiotherapy treatments from different institutions. A predominant role is played by points that represent "the PTV dose". However, for new techniques like Intensity Modulated Radiotherapy (IMRT) - especially step and shoot IMRT - it is difficult to define a point whose dose can be called "characteristic" of the PTV dose distribution. Therefore different volume based methods of reporting of the prescribed dose are in use worldwide. Several of them were compared regarding their usability for IMRT and compatibility with the ICRU Reference Point dose for conformal radiotherapy (CRT) in this study.

**Methods:**

The dose distributions of 45 arbitrarily chosen volumes treated by CRT plans and 57 volumes treated by IMRT plans were used for comparison. Some of the IMRT methods distinguish the planning target volume (PTV) and its central part PTV_x _(PTV minus a margin region of × mm). The reporting of dose prescriptions based on mean and median doses together with the dose to 95% of the considered volume (D_95_) were compared with each other and in respect of a prescription report with the aid of one or several possible ICRU Reference Points. The correlation between all methods was determined using the standard deviation of the ratio of all possible pairs of prescription reports. In addition the effects of boluses and the characteristics of simultaneous integrated boosts (SIB) were examined.

**Results:**

Two types of methods result in a high degree of consistency with the hitherto valid ICRU dose reporting concept: the median dose of the PTV and the mean dose to the central part of the PTV (PTV_x_). The latter is similar to the CTV, if no nested PTVs are used and no patient model surfaces are involved. A reporting of dose prescription using the CTV mean dose tends to overestimate the plateau doses of the lower dose plateaus of SIB plans. PTV_x _provides the possibility to approach biological effects using the standard deviation of the dose within this volume.

**Conclusion:**

The authors advocate reporting the PTV median dose or preferably the mean dose of the central dose plateau PTV_x _as a potential replacement or successor of the ICRU Reference Dose - both usable for CRT and IMRT.

## Background

ICRU 50 and ICRU 62 provide a framework which structures the reporting of external beam radiotherapy treatments from different institutions [1,2]. These reports refer to conventional conformal radiation techniques (CRT). Within that framework, the definition of points that represent "the PTV dose", "prescription dose" or "intended dose" plays a predominant role.

Since then, new techniques like Intensity modulated radiotherapy (IMRT) have been introduced. Early IMRT could only create more inhomogeneous dose distributions, as it was shown by Bratengeier et al. for head and neck studies [3]. Even if today IMRT can be planned more homogeneously, the positioning of a point whose dose can be called "characteristic" of the planning target volume (PTV) is regarded as difficult, if not ambiguous. Therefore the definition of the ICRU Reference Point has become problematic. Previous work like that of Kukolowicz et al. has to be revised for application to IMRT [4]. As a result of the loss of significance of the ICRU Reference Point, a plurality of volume based dose concepts are currently contending, such as the mean dose to the PTV (PTV D_mean_) and the clinical target volume CTV (CTV D_mean_), the dose to 95% of the PTV (PTV D_95_) and others [5-7]. The IMRT Collaborative Working Group recommended the reporting of "Prescribed (intended) dose, as well as the point or volume to which it is prescribed; ....Dose that covers 95% (D95) of the PTV and CTV. Dose that covers 100% (D100) of the PTV and CTV (i.e., the minimal dose). Mean and maximal doses within the PTV and CTV. Percentage of the PTV and CTV that received the prescribed dose (V100)...." [8]. A recent ASTRO recommendation added some further details to be recorded - i.e. D_mean_, D_0_, D_95_, D_100_, V_100 _in PTV and CTV additional to the "prescribed dose" [9].

Often the PTV D_95 _is used as prescribed dose because it is supposed to be a dose prescription regarding biological aspects [7]. This is popular in studies of the Radiation Therapy Oncology Group^® ^(RTOG^®^), i.e. the protocols 0022, 0522, 0615, 0619. This procedure differs from the ICRU Reference Dose concept and the correlation of these two concepts is unclear.

For that reason, the authors examined different volume based definitions. In particular, their consistency with the currently valid "ICRU Reference Dose" (ICRU RD, the dose at the ICRU Reference Point) is investigated. In particular the ratio of the dose defined by several possible ICRU Reference Points and the dose defined by the different reporting procedures is investigated for the same plan. Moreover, the correlation of the pairwise application is explored by calculating the standard deviation of these ratios for all plans and target volumes. Definitions are applied to classical (forward planning) CRT plans and to IMRT-plans. Additionally, simultaneous integrated boost (SIB) IMRT cases were considered, in which nested dose plateaus are formed [10]. To describe the dose to a plateau and to exclude effects of a dose gradient at the border of each volume, the authors preferred to define volumes that are distant to each other. This condition cannot be fulfilled by the clinical target volume (CTV) in the cases of SIB.

## Methods

In this retrospective study, treatment planning was performed on a Philips Pinnacle3™ version 8.0 m planning system (Philips Radiation Oncology Systems, Fitchburg, Wi, USA). Siemens Primus™ (Siemens Healthcare, Erlangen, Germany) and Elekta Synergy™ (with BeamModulator™; Elekta AB, Stockholm, Sweden) linacs were commissioned with 10 mm or 4 mm leaf width (in the isocentre), respectively. The CT slice distance was 3 or 5 mm. A dose grid size of between 2 and 4 mm was chosen. The step and shoot IMRT plans are optimised by the Raysearch™ direct machine parameter optimisation (DMPO) module, a direct aperture optimisation (DAO) method [11]. Not more than 50 segments per plan were used. IMRT plans were irradiated with 7, or (mostly) 9 equidistant beams or 10 non-equidistant fields (breast cases) [12]. The dose distribution was calculated using a collapsed cone algorithm.

The patient data were randomly selected from the normal clinical routine. 70 patients with different tumour localisations and a total of 102 treatment plans were examined. 12 plans resulted from technique changes; 24 plan variants resulted from the application or removal of a bolus. For CRT 38 patients with several localizations were chosen (i.e. 10 head and neck cases, 9 tumours of the abdomen, 7 breast patients with 2 plan each, 4 metastases). 37 patient models with 57 target volumes were used for IMRT techniques (i.e. 19 head and neck patients, 10 breast patients). 6 MV photons were applied for breast, head and neck tumours, 10 MV or 18 MV for the tumours of the abdomen.

### Volume definitions and methods of dose prescription and reporting

All volumes came from clinical practice and were randomly selected. Only one planning target volume was changed for the sake of this study. In addition to the clinical target volume (CTV) and the planning target volume PTV we defined a "PTV_x_" in which the volume is shrunk by an amount × mm, and maintains a distance of × mm towards air. It should be noted that for SIB the nested PTVs abut each other. PTV_x _then excludes the high dose area just as the low dose areas of the PTV. This volume is designated as the "central target volume". It is used to describe the plateau dose. It comprises, depending on the choice of x, approximately the clinical target volume (CTV) in the non-SIB cases. Contrary to the CTV it is designed to contain all the points that eventually would be allowed to be chosen as ICRU dose prescription points; the points from the CTV or PTV from the dose gradient area towards an inner PTV would not comply with that condition. PTV shells that are generated using a margin of less than 2× around an inner target volume would not form a dose plateau and PTV_x _is not defined for the outer PTV ring. This situation will be addressed in the discussion section. For this planning study x = 5 mm was selected, a distance that is frequently chosen to avoid surface effects [13]. In all conventional cases and 22 IMRT-cases only one PTV exists. In 15 IMRT-cases 35 nested target volumes were selected and simultaneously irradiated (SIB) [10]. The target volumes are fundamentally non-overlapping. Therefore, for SIB they abut one another. The extension of the volumes is presented in Table 1.

**Table 1:** Overview

		**Non-breast**					**Breast with bolus**		**Breast without bolus**	
		
		**CRT**	**IMRT**				**CRT**	**IMRT**	**CRT**	**IMRT**
		
				**Single PTV**	**SIB** **Central** **PTV**	**SIB** **Circumferential** **PTV**				
	**n**	**31**	**47**	**12**	**15**	**20**	**14**	**10**	**14**	**10**

Vol [cm^3^]	PTV	837 546	430 471	918 671	124 76	367 212	1240 467	1576 1100	1240 467	1576 1100
	
	PTV_5_	512 383	202 311	522 471	42 37	127 96	797 349	1042 823	797 349	1042 823

σ_D_/D_mean_ [%]	PTV	4.4 2.0	3.9 1.8	4.0 1.5	2.2 0.7	5.0 1.7	3.9 0.9	4.5 1.1	7.0 0.9	9.0 1.3
	
	PTV_5_	2.3 0.7	2.1 0.7	2.1 0.7	1.6 0.6	2.5 0.6	2.9 0.6	2.8 0.7	2.7 0.6	3.1 0.7

D_min_/D_mean_ [%]	PTV	48.4 33.7	51.2 35.0	31.1 37.3	81.9 16.7	40.2 28.7	23.7 24.0	41.4 21.7	0 0	2.5 4.2
	
	PTV_5_	85.8 10.7	88.3 17.6	85.2 21.6	95.4 1.7	84.7 20.6	92.0 2.3	85.7 7.6	86.6 2.3	81.0 4.9

D_max_/D_mean_ [%]	PTV	109.6 4.5	111.9 6.8	109.4 3.2	106.9 2.6	117.0 7.0	111.7 3.0	114.3 3.3	112.3 3.0	116.7 3.0
	
	PTV_5_	108.3 4.3	108.4 4.5	107.4 2.7	104.9 2.1	111.8 4.5	110.3 2.6	112.4 3.5	109.6 2.6	112.4 3.4

n: Number of volumes with related plans. Mean values of Volumes (Vol). Standard deviations of the dose distributions σ_D_, dose minima and maxima (D_min_, D_max_), divided by the mean doses (D_mean_) within PTVs and PTV shrunk by 5 mm (PTV_5_) for several groups of plans (CRT: Conformal radiotherapy, IMRT: Intensity modulated radiotherapy; SIB: Simultaneous Integrated Boost). The upper value in each cell is the mean value; the lower value is the corresponding standard deviation

For breast cases, IMRT was only used to replace CRT if the PTV was extremely curved and standard fields included large lung areas, or if the volumes included mammaria interna lymph nodes. The mean volume for IMRT breast cases was therefore larger than for CRT.

In this study the arithmetic mean and median averages of the dose distribution in the PTV and PTV_5 _were evaluated. In addition D_95 _in PTV and PTV_5 _were determined. Their relationships were calculated for a plurality of ICRU Reference Points selected according to ICRU criteria. For the conventional plans 236 points were used which were acceptable ICRU reference points, for the IMRT plans 340 points. The ICRU Reference Point criteria are: "(1) the dose to the point should be clinically relevant; (2) the point should be easy to define in a clear and unambiguous way; (3) the point should be selected so that the dose can be accurately determined; (4) the point should be in a region where there is no steep dose gradient." [2]. The commission added: "These recommendations will be fulfilled if the ICRU reference point is located: - always at the centre (or in a central part) of the PTV,..."

In almost all cases, four such points are positioned in the central part of each target volume. If possible, one of these points was placed in the centre in the central plane of the PTV. For IMRT plans with single PTVs, additionally the isocentre was chosen as fifth point. The other points were arbitrarily placed in areas which seemed homogeneous. The minimum distance between the points was 1 cm, 0.5 cm for SIB PTVs. The dose to the isocentre and the mean dose of the other four possible ICRU Reference Points were compared.

Furthermore the standard deviations of the doses in the PTV and PTV_5 _were determined. Keeping in mind that D_0 _(= maximum dose) and D_100 _(= minimum dose) can be defective, these values are provided as additional information.

Subgroups of patients are created to allow a cross-check of the data.

### Effects of SIB and surface effects

The characteristics of the 15 head and neck SIB plans were evaluated. These cases were sorted according to their topology: The central PTV and the (one or two) circumferential PTVs.

Surface effects at the patient model surface can drastically change even for a slight change of the outline. The behaviour of the different prescription and reporting methods in such situations was investigated by quantifying the effect of the removal of the bolus for configurations which were initially planned and optimized with bolus. In clinical practice, a bolus can be removed or added according to the skin reaction. The prescription must not change in an other way as the dose to the central points in the PTV (just as for ICRU Reference Points). The breast patients were especially evaluated: Their PTV is near to the patient outline. Thus they are particularly suited to examine surface effects. On the one hand the dose prescription reporting using the PTV and the PTV_x _were compared. On the other hand the influence of using a bolus of 5 mm thickness covering the whole breast was tested both for CRT and IMRT. The bolus was generated by the planning system not considering loose contact to the skin as often can be observed in clinical practice. Hence, in the breast group two extremes are compared, because in clinical practice neither such a perfect bolus is available nor would the cases with skin involvement be irradiated without a bolus.

## Results

### Plan quality parameters

The relative standard deviation of the doses in the PTV (PTV_5_, respectively) was 4.4% (2.3%) for the CRT non-breast plans, 7.0% (2.7%) for the breast plans without bolus and 3.9% (2.9%) for the same plans with a bolus over the whole breast (see also Table 1). The influence of the surface on the PTV standard deviation can clearly be seen, whereas the standard deviation of PTV_5 _is not affected. For IMRT, the relative standard deviation of the dose in the PTV (PTV_5_) was 3.9% (2.1%) for the non-breast plans, 9.0% (3.1%) for the breast plans without bolus and 4.5% (2.8%) for the same plans with bolus (Table 1). This result is similar to that for the CRT-plans, indicating that for step and shoot IMRT using DMPO similar dose homogeneity could be achieved as for the CRT plans, although the PTV shape was more complex. A detailed view of the IMRT results shows differences for the inner PTV (σ_D _= 2.2% (1.6%)) and the annular PTV shells (σ_D _= 5.0% (2.5%)). For the latter, the standard deviation and hence dose homogeneity suffers especially in the PTV from the additional dose gradient towards the inner target volumes. These findings were similar, if CTV was used instead of PTV_x _for nested volumes: the standard deviation increased by a factor of 1.5, (for 3 of 26 volumes by more than a factor of 2; details see below).

The minimum doses for CRT were around 31% (86%) in relation to the prescription dose, for IMRT 44% (86%) with large standard deviations of 33% (10%) and 34% (16%), respectively. (not shown in the tables). However, these results can largely be influenced by PTV delineation, surface effects, grid size and dose calculation algorithm.

The isocentres in the single PTV IMRT cases were used to control the adequate setting of the arbitrary chosen ICRU reference points. The mean value of their doses differed by a factor of 0.9995 and the standard deviations were 2.2% and 2.4%, respectively. This indicates a reasonable ICRU Reference Point positioning in this work.

### Comparison of prescription and reporting methods

Table 2 correlates some volume based prescription and reporting methods and a selection of allowed ICRU Reference Points with an ICRU Reference Dose (RD) for non-breast plans. The first row of each cell is the ratio of the method of a column and to that of a row, averaged over all cases. In the second row the respective standard deviation of this average process is presented which indicates the dispersion of the data. Ratios of the reported dose for an identical dose distribution can be compared using the upper and the lower part of the table for CRT and IMRT, respectively. ICRU RD (case-mean) is the dose to the mean value of all chosen examples of an ICRU Reference Point of each case, finally averaged over all cases. In the right column, ICRU RD, the average of all normalized ICRU Dose Points of all cases is presented to show the statistical dispersion if different single points are used to represent a dose distribution.

**Table 2:** Correlation of prescriptions (non-breast cases)

	**denominator\numerator**	**PTV** **D_Mean_**	**PTV_5_** **D_Mean_**	**PTV** **D_95_**	**PTV_5_** **D_95_**	**ICRU** **RD** **(Case-Mean)**	**ICRU** **RD**
	
		**[%]**	**[%]**	**[%]**	**[%]**	**[%]**	**[%]**
CRT n = 35	PTV D_Median_	99.5 0.4	100.4 0.6	92.4 2.9	96.6 1.0	99.9 1.4	**99.9** **1.8**
	
	PTV D_Mean_		100.9 0.7	92.8 2.9	97.1 1.2	100.5 1.7	**100.5** **2.0**
	
	PTV_5 _D_Mean_			92.0 3.1	96.2 1.0	99.5 1.5	**99.5** **1.8**
	
	PTV D_95_				104.7 3.1	108.3 4.0	**108.0** **4.2**
	
	PTV_5 _D_95_					103.5 1.8	**103.4** **2.1**
	
	ICRU RD (Case Mean)						**100.0** **1.1**

IMRT n = 47	PTV D_Median_	100.0 0.8	100.5 1.0	94.7 2.3	97.2 1.5	100.3 1.7	**100.3** **2.7**
	
	PTV D_Mean_		100.5 1.5	94.7 2.4	97.3 1.9	100.3 2.1	**100.4** **3.0**
	
	PTV_5 _D_Mean_			94.3 2.2	96.8 1.1	99.7 1.3	**99.7** **2.4**
	
	PTV D_95_				102.6 2.0	107.0 2.8	**107.0** **3.5**
	
	PTV_5 _D_95_					103.0 1.8	**103.0** **2.7**
	
	ICRU RD (Case Mean)						**100.0** **2.1**

Correlation of several prescription and reporting methods. All methods report for the same dose distribution per study. -- Non-breast cases. The upper value in each cell is the mean value; the lower value is the corresponding standard deviation. ICRU RD: ICRU Reference Dose; PTV_5_: PTV shrunk by 5 mm; Case mean: Mean value of four (IMRT with a single PTV: five) points suitable for dose description according to ICRU 50/62

The standard deviation of the ratio ICRU RD/ICRU RD (case-mean) - last row, right column - is a measure of the statistical dispersion of the dose at the chosen ICRU Reference Points within a volume, a measure of the correlation among the chosen points. This value should be improved upon by any method which competes with the point based methods. Standard deviations of 1.3% and 2.3% for the ICRU RD point to point correlations are found for all CRT plans and all IMRT plans, respectively (not shown in the tables). They should also be considered as benchmarks for the correlation of the ICRU RD with any other reporting method: the standard deviations over all plans were 1.5% and 1.4% for PTV_5 _D_mean_, 1.6% and 1.8% for PTV D_median_, 1.9% and 2.6% for PTV D_mean_, 1.7% and 2.2% for PTV_5 _D_95_, 4.3% and 5.9% for PTV D_95 _(CRT and IMRT, respectively). For the first three reporting methods, the average quotient with the reporting using the ICRU Reference Point was biased by less than 0.6% (when using all CRT and IMRT plans), whereas the quotient for PTV_5 _D_95 _was 96% and for PTV D_95 _92%. The D_95 _values should be compared with an independent evaluation in the author's clinic over 350 patients: there a value of 94.3% for a mixture of both PTV groups was achieved.

A cross-check of dose reporting concepts for the breast cases (with bolus; Table 3) and for non-breast, single PTV IMRT (Table 4) reveals almost the same results. Only the dose was slightly more homogeneous for single PTV IMRT (Table 1). Consequently, the correlation of one ICRU Reference Point with the mean value of all possible ICRU Reference Points expressed by the standard deviation was 2.0% for non breast IMRT in a single PTV (not shown in the tables) compared with 2.6% for breast IMRT (Table 3).

**Table 3:** Correlation of prescriptions (breast cases -- surface effects)

		**denominator\numerator**	**PTV** **D_Mean_**	**PTV_5_** **D_Mean_**	**PTV** **D_95_**	**PTV_5_** **D_95_**	**ICRU** **RD** **(Case Mean)**	**ICRU** **RD**
	
			**[%]**	**[%]**	**[%]**	**[%]**	**[%]**	**[%]**
with bolus	CRT n = 14	PTV D_Median_	99.9 0.3	100.0 0.4	94.1 1.5	95.5 1.4	98.5 1.5	**98.5** **1.9**
		
		PTV D_Mean_		100.1 0.3	94.1 1.3	95.6 1.3	98.6 1.4	**98.6** **1.8**
		
		PTV_5 _D_Mean_			94.1 1.3	95.5 1.1	98.5 1.3	**98.5** **1.8**
		
		PTV D_95_				101.6 1.2	104.7 1.8	**104.8** **2.2**
		
		PTV_5 _D_95_					103.1 1.6	**103.2** **2.0**
		
		ICRU RD (Case Mean)						**100.0** **1.3**
	
	IMRT n = 10	PTV D_Median_	99.5 0.3	100.9 0.3	92.1 2.2	96.4 1.0	100.7 1.1	**100.7** **2.8**
		
		PTV D_Mean_		101.4 0.5	92.5 2.0	96.9 1.1	101.2 1.0	**101.2** **2.2**
		
		PTV_5 _D_Mean_			91.3 2.3	95.6 1.1	99.9 1.0	**99.9** **2.7**
		
		PTV D_95_				104.7 2.2	109.5 2.6	**109.5** **3.6**
		
		PTV_5 _D_95_					104.5 1.7	**104.5** **3.1**
		
		ICRU RD (Case Mean)						**100.0** **2.5**

without bolus	CRT n = 14	PTV D_Median_	98.8 0.3	100.6 0.4	88.0 1.5	96.4 1.4	99.3 1.5	**99.3** **1.9**
		
		PTV D_Mean_		101.8 0.3	89.0 1.3	97.5 1.3	100.5 1.4	**100.4** **1.8**
		
		PTV_5 _D_Mean_			87.5 1.3	95.8 1.1	98.7 1.3	**98.7** **1.8**
		
		PTV D_95_				109.7 1.2	113.0 1.8	**112.7** **2.2**
		
		PTV_5 _D_95_					103.0 1.6	**103.1** **2.0**
		
		ICRU RD (Case Mean)						**100.0** **1.3**
	
	IMRT n = 10	PTV D_Median_	97.7 0.7	101.4 1.0	81.5 3.2	96.3 1.4	102.1 1.0	**102.1** **2.6**
		
		PTV D_Mean_		103.8 1.4	83.5 2.9	98.6 1.9	104.6 1.5	**104.6** **2.9**
		
		PTV_5 _D_Mean_			80.4 3.8	95.0 1.0	100.7 0.8	**100.7** **2.5**
		
		PTV D_95_				118.3 6.4	125.5 6.1	**125.5** **6.6**
		
		PTV_5 _D_95_					106.1 1.5	**106.1** **3.0**
		
		ICRU RD (Case Mean)						**100.0** **2.4**

Correlation of several prescription and reporting methods. All methods report for the same dose distribution per study. -- Breast cases with and without bolus (upper and lower part, respectively). The upper value in each cell is the mean value; the lower value is the corresponding standard deviation. ICRU RD: ICRU Reference Dose; PTV_5_: PTV shrunk by 5 mm; Case mean: Mean value of four (IMRT with a single PTV: five) points suitable for dose description according to ICRU 50/62

For non-breast plans the reporting using PTV D_median_, PTV D_mean _and PTV_5 _D_mean _led to comparable results with respect to the mean of the ICRU Reference Doses. The larger standard deviations for the ICRU RD reflect the fluctuation due to the choice of the position of the ICRU Reference Point. The results for CRT and IMRT are quite similar.

D_min _was not presented in the tables because the standard deviation of the correlation to other methods was always above 10% for D_min _of PTV_5 _and even exceeded 30% for D_min _of PTV.

Detailed data for subgroups of non-breast IMRT are shown in Table 4. Here 12 patients with a single PTV are differentiated from patients with SIB. For the latter, the 15 central volumes and the 20 circumferential volumes were distinguished. Only volumes with distances of at least 5 mm to the patient model outline or plans with boluses were considered.

**Table 4:** Correlation of prescriptions (non-breast IMRT subgroups - topological aspects)

	**denominator\numerator**	**PTV** **D_Mean_**	**PTV_5_** **D_Mean_**	**PTV** **D_95_**	**ICRU** **RD** **(Case-Mean)**	**ICRU** **RD**
	
		**[%]**	**[%]**	**[%]**	**[%]**	**[%]**
Single PTV n = 12	PTV D_Median_	99.5 0.3	100.7 0.4	94.1 1.9	100.6 1.3	**100.6** **2.4**
	
	PTV D_Mean_		101.2 0.6	94.6 1.8	101.1 1.4	**101.1** **2.5**
	
	PTV_5 _D_Mean_			93.4 2.1	99.9 1.3	**99.9** **2.4**
	
	PTV D_95_				107.0 2.8	**107.0** **3.5**

Central PTV n = 15	PTV D_Median_	100.0 0.3	101.0 0.8	96.5 0.7	100.4 0.8	**100.4** **1.6**
	
	PTV D_Mean_		100.9 0.8	96.5 0.8	100.4 0.9	**100.4** **1.6**
	
	PTV_5 _D_Mean_			95.6 1.3	99.4 0.8	**99.4** **1.6**
	
	PTV D_95_				104.0 1.4	**104.0** **2.0**

Circumferential PTV n = 20	PTV D_Median_	99.9 1.3	100.0 1.2	93.6 2.4	100.0 2.2	**100.0** **3.4**
	
	PTV D_Mean_		99.8 2.0	93.4 2.7	99.8 2.9	**99.8** **3.9**
	
	PTV_5 _D_Mean_			93.8 2.4	99.7 1.6	**99.7** **2.9**
	
	PTV D_95_				106.9 4.2	**106.9** **5.0**

Correlation of several prescription and reporting methods for subgroups of IMRT plans (non-breast cases) without surface effects (with bolus and PTV-distance to patient outline > 5 mm). All methods report for the same dose distribution per study. The upper value in each cell is the mean value; the lower value is the corresponding standard deviation. ICRU RD: ICRU Reference Dose; PTV_5_: PTV shrunk by 5 mm; Case mean: Mean value of four (IMRT with a single PTV: five) points suitable for dose description according to ICRU 50/62. Central and circumferential volumes together form the volumes of SIB (2 or 3 nested volumes)

For SIB IMRT, the dose ratio (PTV_5 _mean dose) of the outer to the adjacent inner volume was 0.89 (0.82 up to 0.93) for the cases with 2 volumes, 0.87 (0.78 up to 0.92) for cases with 3 nested volumes (outer volume pair) and 0.96 (0.94 ... 0.99) (inner volume pair). Comparing the standard deviations of PTV_5 _and CTV for the related outer volumes, the standard deviation of the dose distributions increased for the CTV by a factor of 1.49, 1.86 and 1.08, respectively. The mean dose to the CTV increased with respect of the mean dose to the PTV_5 _was by a factor of 1.018, 1.019 and 1.005, respectively. Selecting the volume pairs with PTV_5 _mean dose differences of more than 9% (10% up to 22%) between inner and outer PTV, led to CTV/PTV_5 _dose ratios of 1.029; the ratio of the CTV/PTV_5 _standard deviations was 2.11 (1.68 to 2.89), respectively.

**Figure 1:**
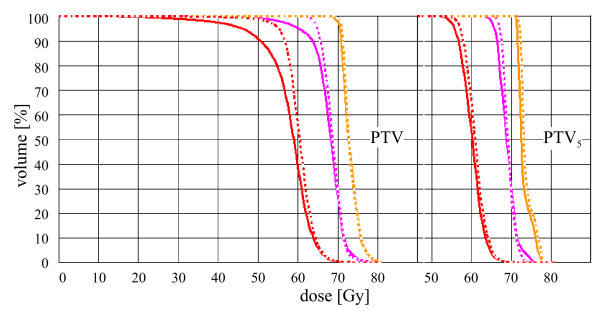
**Example of a head and neck IMRT case (not used for quantitative evaluation) with three adjacent, nested targets, partially abutting the patient outline**. DVH for irradiation with 6 MV photons and bolus (thickness 5 mm): dashed line. Without bolus: solid line. Left diagram: Three nested, adjacent, non-overlapping PTV. Right diagram: Three nested PTV_5 _(PTV shrunk by 5 mm). From left to right: Outer (circumferential) to inner (central) PTV.

Table 3 presents the planning results of the breast cases (CRT: 14 cases; IMRT: 10 cases). The upper part comprises the cases with 5 mm boluses, whereas the lower part represents the same cases without a bolus. This table demonstrates the effect of the extended near-surface areas as typical for breast patients (the PTV is delineated approaching the patient outline). Similar results were achieved if the bolus for five non-breast cases was removed (not shown here, see Fig. 1).

## Discussion

### Comparison with other published results

Das et al. compared the IMRT practice of five institutions with differing planning systems [5]. 803 brain, head and neck and prostate cancer patient plans were evaluated, with patient group characteristics similar to the non-breast patient group of this work. The prescription dose had been correlated with D_min_, D_max_, D_median _and the dose to the isocentre. Except the median dose all parameters showed only weak correlation to the prescription dose. These results agree with the results of this work. In Das' work, the standard deviation of the ratio of prescription dose and median dose as a measure of the correlation can be estimated to be between 2% and 3%. In this work, the standard deviation of the ratio ICRU RD (single point) and D_median _was 1.8% for CRT and 2.7% for IMRT plans of the non-breast cases (Table 2). The latter value was mainly influenced by SIB cases with onion-skin-like (nested) PTVs (3.4%). Otherwise the standard deviation was 2.4% (single PTV) and 1.6% (central PTV in a SIB constellation - see Table 4). Thus, the results are similar.

Yaparpalvi et al. examined the IMRT plans of 117 patients, some of them with 3 different IMRT plans [7]. They compared three prescription and reporting methods: the site specific RTOG guideline, ICRU RD and D_mean_. Their results showed a strong correlation of D_mean _and ICRU RD with an estimated *σ*_D _of roughly 2% (from Yaparpalvi Fig. 1) and a much weaker correlation of both with the D_95_, D_97_, D_98_-prescriptions of several RTOG protocols. The ratio of prescription dose due to the RTOG guidelines and the ICRU RD was between 103.6% (RTOG 0418, D_97_) and 105.1% (RTOG 0022, D_95_); the latter should be compared with the non-breast cases of this work (107.0% for all non-breast IMRT cases; 107.0% and 106.9% for single and circumferential volumes, 104.0% for the central volume of a SIB). They also concluded that the D_median _in the PTV would be a better representation of the ICRU RD than the D_mean _agrees with the results of this work.

Several meeting contributions have addressed future ICRU recommendations on dose prescription, recording and reporting [14,15]: Single point prescription and reporting will be given up in favour of volume based methods. It was announced that the median dose would play a prominent role. This is supported by this work, although PTV_x _could be a concept of more biological relevance, in combination with the related standard deviation in this volume (see below).

### The use of PTV D_95_

**Figure 2:**
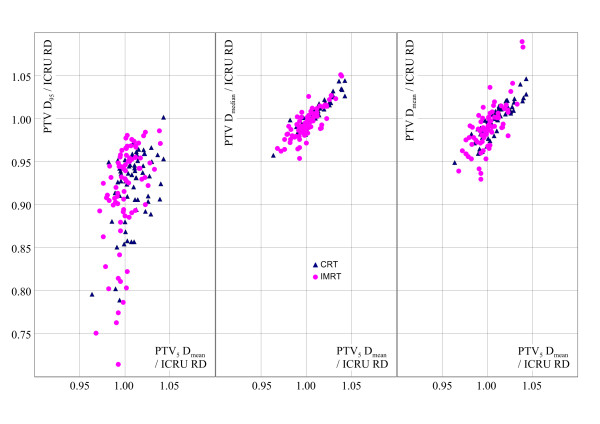
**Correlation of several prescription and reporting methods (all related to ICRU RD, the mean value of 4 or 5 points fulfilling the ICRU 52/60 Reference Dose criteria) with the ratio of the prescriptions PTV_5 _D_mean_/ICRU RD (PTV_5 _D_mean_: mean dose for the central part of the PTV)**. Results for all plans of the study. All methods report for the same dose distribution per study. Circles: All IMRT plans and related volumes. Triangles: All CRT plans used in this study. left: PTV_5_D_mean _vs. PTV D_95 _(dose to 95% of the PTV). middle: PTV_5_D_mean _vs. PTV D_median_. (median dose for the PTV) right: PTV_5_D_mean _vs. PTV D_mean _(mean dose of the PTV).

The use of D_95 _as a substitute or successor for the ICRU RD would lead to a conversion factor of typical 1.08 ± 0.04 between PTV D_95 _and ICRU RD (non-breast plans, Table 2). Such a factor ought to be considered, if the prescription specification is changed. i.e. using PTV D_95 _instead of PTV D_mean _without adequate correction of the prescribed dose would lead to a dose escalation. However, because of the weakness of the correlation - expressed by the standard deviation of 2.8 to 4% (see Fig. 2) - such a transformation cannot be recommended in general. D_95 _is always only weakly correlated to the ICRU RD for both, PTV_5 _and particularly for PTV - in contrast to other methods (see Fig. 2b). Even compared with the single point - ICRU RD Point correlation (with its standard deviation of 2% - 2.5% for IMRT plans) it is more loosely correlated with former ICRU RD. The conversion of a D_95 _prescription would also be greatly affected by surface effects, as can be seen for the CRT and IMRT breast cases (varying from 1.04 to 1.25 in Table 3 and similar results for the outer SIB volume in Table 4).

To compare IMRT results with earlier CRT results and to assure continuity with respect to former dose prescription, another substitute for ICRU RD must be provided. PTV D_95 _and PTV D_min _(or D_01 _...) may be reported as additional information to describe the homogeneity of the dose in the PTV. It should be noted that neither the dose below the D_95 _is restricted to the peripheral PTV areas nor is the depth of a drastic dose reduction below the D_95 _restricted by using this prescription and reporting method. Therefore, usage of D_95 _alone, can neither guarantee a certain lower limit for a tumour control probability nor "an expected clinical outcome of the treatment" [1]. Dose prescription and description of the plan quality cannot be achieved with a single parameter. An ASTRO/AAPM working group recommends three DVH-points to describe biologically relevant PTV-data of a dose distribution [16]. Two of the points form the lower and upper dose limits, the third point should provide the dose "that covers the target" [16]. However, also mean and median doses in the PTV or PTV_x _seem to be appropriate candidates to describe the "typical" dose, some of them much more closely correlated to the ICRU RD, thereby making CRT and IMRT plans more comparable.

Moreover, for the breast cases (largest standard deviation relative to the ICRU RD; Table 3) and for some SIB in the circumferential PTV (not shown in detail), the D_95 _prescription depends largely on surface effects (i.e. changes of more than 5% for an irradiation with or without a bolus). The exemplary DVH of a patient with three concentric head and neck target volumes in Fig. 1 depicts the same problem. Application of a 5 mm bolus changes the course of the PTV curve drastically at the low dose limb of the DVH. Obviously, minor changes in the placement of the bolus would influence a prescription based on D_95 _of the PTV, although only the peripheral PTV areas are affected. Similarly, D_95 _depends clearly on further parameters. Central volumes in our clinical practice tend to have much lower D_95 _to ICRU RD ratios (1.040%, see Table 4) in contrast to 107.0% and 106.9% for single or circumferential PTVs. A prescription and reporting based on central areas (CTV, PTV_x_) would be much more insensitive with respect to effects of surface and volume delineation variations.

This article is not intended to determine whether a D_95_(PTV) or a D_mean_(PTV_x_) prescription would be the better method to prescribe tumour control. Both require more information about the low dose parts in relevant areas that limit the tumour control probability (TCP) and hot spots that increase the probability of irreversible damage to healthy tissue. The D_mean_(PTV_x_) approach implies additional information on the local behaviour of the dose distribution that is lost in the D_95 _concept: as can be seen below, D_mean_(PTV_x_) together with additional information like the standard deviation of the dose distribution allows linkage to a more biologically based evaluation (see last section).

### Dose fluctuations in the target

The fluctuations of the ICRU RD increase for IMRT as predicted by several authors [7]: the standard deviation for the ICRU RD is slightly larger for IMRT plans than for the CRT techniques, and all correlations of other methods are weaker for IMRT than for CRT (the standard deviation of the quotient of the reported results is larger). However, it should be noted, that the standard deviations of the mean doses in the PTV and the PTV_x _(σ_D_/D_mean _in Table 1) are comparable for single PTV IMRT and CRT plans. This means that the fluctuations of the dose in PTV or PTV_5 _as a whole are comparable for CRT and this special type of IMRT (IMRT based on the DMPO optimization). Conversely, the standard deviations of the ICRU RD from several chosen points tend to be smaller for CRT plans than for IMRT plans ("PTV D_mean_" and "PTV_5 _D_mean_" in Table 2, 3 and 4, last column "ICRU RD"). Perhaps this can be interpreted as if dose fluctuations of classical CRT plans were less concentrated in the areas that were typically chosen for ICRU Reference Points. This underlines the requirement of a volume integrating prescription and reporting method for IMRT, even for rather homogeneous IMRT plan types as used in this work.

It should be noted that the homogeneity of IMRT plans has continuously increased in the past few years. For head and neck as well as related cases an extensive exploration of data from the first IMRT decade had been performed [3]. Published DVHs (between 1990 and 1998) for realistic cases including scatter and absorption σ_D _was 3.3% up to 11% of a target with more than 5 mm to the patient outline, the related mean value of comparable non-IMRT rotational techniques was 3.1%, classical opposed fields with electrons reached 6% which should be compared to a mean of 2.2% for σ_D _of the dose in PTV_5 _which are reached for DMPO in head and neck cases in this work. Sliding window or volumetric arc techniques should be able to create even more homogeneous dose distributions. These results also encourage the use of "non-D_95_" plans, but prescription and reporting methods with a conversion factor around 1.00 in relation to the hitherto valid ICRU RD, if CRT and IMRT plans should be compared.

### SIB and surface effects of the PTV and CTV mean dose

The mean dose to the CTV for the outer SIB volumes overestimated the plateau dose by 2% (in some cases almost 4%). For these outer PTVs of SIB, the dose gradient towards the inner PTVs influences the mean dose of the outer PTV. It raises the mean dose in the CTV, pretending a higher dose as actually reached in the dose plateau, whereas the dose overkill near the inner gradient probably cannot compensate a potential underdosage at the plateau.

This effect depends clearly on the dose difference of inner and outer volumes and could be relevant for dose differences of 10% and more; the overestimation of the plateau dose could exceed 3% if the CTV mean dose would be used.

For PTV D_mean _an underdose to the peripheral areas and an overdose to the inner areas could compensate each other. But this effect depends on the geometry of each individual case, as can be deduced from the higher standard deviations of PTV D_mean _(Table 4, circumferential volumes), indicating overestimations and underestimations that compensate each other averaging PTV D_mean _over all patients. PTV_x _avoids both problems.

SIB with nested volumes with a thickness of less than or equal 2× form no dose plateau in the outer PTV (such volumes were not addressed within this work). These SIB cases cannot be described by a concept equivalent to ICRU points, because no point can be found which would be representative for this volume. Such volumes are mostly described by their minimal dose or concepts like D_99_, D_98 _etcetera.

Adding a bolus to a breast plan changes the PTV mean dose with respect to the ICRU reference dose by 2%. This is due to surface effects that should actually not influence the prescription, which should be based on the dose within the central dose plateau with the highest accumulation of tumour cells. Furthermore, dose calculation algorithms tend to create erroneous results at the patient surface. Obviously a dose of 0% as can be seen in table 1 for CRT breast cases is absurd. This topic will not be addressed here in detail, but clearly such areas should be omitted when important values as the prescription dose are to be determined. Similar changes of the PTV mean dose can be expected due to delineation effects of the PTV shape [17,18]. The same observation can be made in the example from Fig. 1: mean values of the central plateau of each target (PTV_5_) are not affected by using a bolus or not (right side), whereas manifestly the mean dose of the PTV itself significantly changes.

In non-SIB cases PTV_x _could resemble CTV, which then could be alternatively used for prescription and reporting. However, CTVs with points near the surface should be chosen with caution. As can be seen in the case of the tissue of the mammary gland for slender patients, CTV sometimes approaches the outline more closely than 5 mm. The choice of 5 mm is due to the fact that these 5 mm often are used in daily practice (i.e. Fogliata 2005) [13].

In summary, only median dose in the PTV and the mean dose to the PTV_x _remain in contention to be the worthy successor of the ICRU RD. Both were mutually strongly correlated (standard deviation of the quotient of about 1%) and could be converted using a factor of 1.00.

### Additional advantages and disadvantages of the mean dose in PTV_x_

The definition of PTV_x _includes the set of all points which can be chosen as ICRU Reference Points as a subset. Perhaps, PTV_x _could even be interpreted as the set of all points that could possibly be chosen as ICRU Reference Points. Therefore, the close correlation to the special choice of ICRU Reference Points in this work is not surprising. The x = 5 mm margin ensures that all points are within a dose plateau and not near to the steep dose gradient at the borders of the PTV. In several cases, PTV_5 _includes about 58% (32% to 73%) of the PTV volume for the CRT and 41% (7% to 77%) for the IMRT cases. Furthermore, all the excluded voxels are supposed to have a lower tumour cell density than the centre of the PTV, which includes the CTV. Due to the mean value theorem for integration, the mean value of PTV_x _can be represented by one or more points within the plateau PTV_x _of the PTV. This is not necessarily so for a D_95_prescription - not even in the majority of cases. If a prescription point ought to be defined, it should be placed at the border of the PTV near to the steep dose gradients. (Only for IMRT plans with dose gaps in the centre of the PTV- that is for bad plans - a reference dose point representing the prescription dose could be placed somewhere in the central PTV area.)

In contrast to the prescription and reporting based on the median dose which is regarded as a relevant method, the mean value of PTV_x _could be a base for later biological interpretations with diverse biological models [5,14]. Brahme demonstrated that the pair of mean value and standard deviation σ_D _of the dose in a volume with constant tumour cell density provides the possibility of the subsequent approximate recalculation of tumour control probabilities or equivalent doses with arbitrary biological models [19]. (PTV_x _is a better approximation of such a volume than PTV with its much smaller tumour cell densities in the periphery.) Bleher et al. calculated a σ_D _correction of the tumour control probability for a known "homogeneous dose" probability curve TCP(D) [20]:

(1a)

The first non-trivial non-zero term of a Taylor-series around D = D_mean _(PTV_x_) with

(1b)

The definition of PTV_x _additionally allows the evaluation of D_min _of the dose in the central PTV area - PTV_x _- and the standard deviation *σ*_D _of the dose therein. Both are useful to control the dose homogeneity in the most important part of the PTV. For the whole PTV, potential dose inhomogeneities in the centre are covered by the dominating dose inhomogeneities at the periphery, which is caused by uncertainties of the PTV definition or uncertainties in the dose calculation (dose grid, surface effects). Therefore, D_min _and σ_D _of the PTV are less meaningful than D_min _and σ_D _of the CTV or PTV_x_. Such information - PTV_x _D_min _and σ_D _- is routinely used in our clinic for automatic control of these aspects of plan quality. In our institution, σ_D _< 3.3% is striven for to avoid relevant TCP-reductions due to inhomogeneity. For *γ *= 3 (the steepness of TCP-curve) and σ_D _< 3.5% Brahme estimated a decrease of 5% for the TCP.

An additional advantage of the mean dose in the PTV_x _is the additivity of mean doses in contrast to median doses or D_95_, albeit the biologically equivalent doses cannot simply be summed up.

Some limitations of the D_mean_(PTV_x_) concept should not be concealed. For example for stereotactic treatments, dose inhomogeneity may be intended. This inhomogeneity is not arbitrary. The hot spots for small volumes are preferably in the central CTV. Commonly minimal doses (D_100-*y *_with small y) in the CTV and PTV together with maximum doses (D_z _with small z) are reported. All these prescription values are relevant. Nevertheless even in the case of stereotactic irradiations, a wider plateau with steep gradient near the PTV edge and a sharply peaked dose distribution with less steep gradient could be delivered with the same specific data as stated above, although the former dose distribution would obviously provide the better TCP. The additional use of D_mean_(PTV_x_) would reveal the differences of both plans.

As a further disadvantage it should be noted that PTV_x _is currently not in usage (as long as it is not chosen identical with the CTV). Additionally the choice of x (x = 5 mm in this work) is arbitrary.

If the usage of PTV_x _is considered to be too arbitrary, a "modified CTV" could be used as a compromise: A CTV reduced by margins to other CTVs in the vicinity.

## Conclusion

"The dose to the patient", formerly represented by the ICRU Reference Point, continues to play a prominent role in daily practice (i.e. the doctor's letter). In contrast the intended additional values in ICRU 62 [2], the expanded framework of these recommendations, sometimes tend to be in the background - all the more reason that a careful and coherent definition of this dose term is performed.

As successor for the ICRU Reference Point, equally usable for IMRT and CRT, the authors recommend the median dose to the PTV or - preferably - the mean dose to the PTV_x_, the central plateau. Both are "near" to the physical dose distribution and provide a consistent extension of the ICRU Reference Dose (strong correlation and conversion factor ≈ 1.00). Mean doses to CTV and PTV do not to such an extent. Usage of PTV_x _D_mean _adds the possibility of using of the standard deviation in the PTV_x _for later evaluation of tumour control probabilities. Moreover it provides further parameters, which control the homogeneity of the target (like standard deviation and minimal dose to the central plateau).

## Competing interests

The authors declare that they have no competing interests.

## Authors' contributions

KB was responsible for the primary concept and the design of the study; he compiled the results and drafted the manuscript; MO evaluated most of the results; MG critically accompanied the study and revised the manuscript; MF was responsible for the patients, reviewed patient data and revised the manuscript. All authors read and approved the final manuscript.
